# Mediating effect of lower extremity muscle strength on the relationship between mobility and cognitive function in Chinese older adults: A cross-sectional study

**DOI:** 10.3389/fnagi.2022.984075

**Published:** 2022-11-03

**Authors:** Yaoxin Chen, Yijun Zhan, Hong Wang, Hui Zhang, Yiwen Cai, Liaoyao Wang, Wenyan Zhu, Haiyue Shen, Jian Pei

**Affiliations:** ^1^Department of Acupuncture, Longhua Hospital, Shanghai University of Traditional Chinese Medicine, Shanghai, China; ^2^College of Rehabilitation Sciences, Shanghai University of Medicine and Health Sciences, Shanghai, China; ^3^Shanghai University of Medicine and Health Science Affiliated First Rehabilitation Hospital, Shanghai, China

**Keywords:** aging, cognitive function, mediation, mobility, muscle strength

## Abstract

Aging is a multifactorial process associated with irreversible decline in mobility and cognitive function. However, the mechanisms underlying the relationship between mobility and cognitive function remain elusive. In specific, the mediating effect of muscle strength, which is essential to maintain mobility, on this relationship has yet to be clarified. Accordingly, we performed a cross-sectional study involving Chinese older adults to understand the role of muscle strength in the relationship between mobility and cognitive function. The cognitive function and physical performance of 657 community-dwelling participants aged over 65 years old were observed. Cognitive function was assessed using the Mini-Mental State Examination, whereas physical performance, including mobility and muscle strength, was measured *via* Timed Up-and-Go Test and knee extension strength measurement. Data were statistically analyzed using PROCESS Model 4 developed by Hayes, and 595 complete data were finally included. Physical performance (mobility and muscle strength) was significantly correlated with cognitive function (*p* < 0.01). Muscle strength was negatively correlated with mobility (*r* = −0.273, *p* < 0.001) and positively correlated with cognitive function (*r* = 0.145, *p* < 0.001). Muscle strength accounted for 20.1% of the total mediating effects on the relationship between mobility and cognitive function, which revealed the partial mediating role of lower extremity muscle strength in this relationship.

## Introduction

Although physical disability and cognitive impairment have been studied as separate entities, concurrent physical and cognitive decline in later life can synergistically lead to adverse outcomes (Yu et al., 2017; Montero-Odasso et al., 2019). In the past decade, some operational definitions have been proposed to consider the concurrent physical and cognitive decline condition, including the terms of “cognitive frailty” (Panza et al., 2018) and “motoric cognitive risk syndrome (MCR)” (Verghese et al., 2013). MCR is a predementia syndrome characterized by subjective cognitive concerns and slow gait, which is a highly prevalent condition associated with a significant increase in risk of all-cause mortality in multiple countries and populations (Chhetri et al., 2020; Bortone et al., 2021, 2022). However, the underlying mechanism remains unclear. Cumulating evidence demonstrates that aging not only leads to structural alterations in individual components of the neuro-muscular system but also a loss of complexity and dedifferentiation (become common to different functions) at the brain, muscle, and behavioral levels, with systematic reorganization of interactions within and between different levels and functional domains (Sleimen-Malkoun et al., 2014). Thus, a common underlying pathophysiological process possibly contributes to the cognition and mobility domains in the brain, such as the cerebellum and hippocampus (Liu et al., 2020), for more widespread cortical activation and connection involved in cognitive and motor tasks in older adults than younger ones (Coppi et al., 2014; Qin and Basak, 2020).

Mobility is a crucial part of physical performance that requires certain degrees of flexibility, balance, and muscle strength. Muscle strength diminishes much earlier than mobility (Chen and Arai, 2020; Lunt et al., 2021), and decreased muscle strength often leads to mobility problems (Winger et al., 2021). Predictive models that consider associations between multiple covariates and cognitive function include mobility as a predictor, suggesting that interventions slow down adverse trajectories of cognitive decline by acting on potentially modifiable factors, such as improving muscle strength (Hartley et al., 2021). Reduced muscle strength is associated with an increased risk of all-cause mortality in older adults (Teunissen et al., 2022). Evidence from longitudinal studies shows that decreasing muscle strength is associated with cognitive decline (Kim et al., 2021). Increasing muscle strength has been an important treatment target in patients with cognitive impairment (Bossers et al., 2015). Given its importance in maintaining mobility, muscle strength might exert an important mediating effect on the relationship between mobility and cognitive function.

Compared with upper extremity grip strength, lower extremity muscle measures are more strongly and directly associated with the assessment of mobility (Winger et al., 2021). To date, few studies analyzed the mediating role of lower extremity muscle strength in the relationship between mobility and cognitive function in older adults. Understanding the specific role of lower extremity muscle strength in the relationship between mobility and cognitive function may help elucidate the mechanisms underlying this relationship. Thus, the present study aimed to investigate the mediating effect of lower extremity muscle strength on the relationship between mobility and cognitive function.

## Materials and methods

### Participants

Residents of Chongming District, Shanghai, China who had joined China’s national free physical examination program between August and November 2018 were enrolled in this study. Inclusion criteria were as follows: (I) older adults ≥65 years old; (II) have lived in the area for at least 1 year; and (III) willingness to join the study. Exclusion criteria were as follows: (I) failure to complete interviews due to severe visual and hearing impairment; (II) inability to perform the lower extremity muscle strength test or the TUGT; and (III) having a diagnosis that could affect muscle strength or mobility. Of the 657 participants for whose questionnaires were available, 62 with missing covariates were excluded. A final sample of 595 participants remained for current analyses. The participants were fully informed about the nature of the study and signed an informed consent form. This study was approved by the ethics committee of Shanghai University of Medicine and Health Sciences and conducted in accordance with the principles of the Declaration of Helsinki.

### Assessment of cognitive function

Cognitive assessment was completed by trained investigators using the Mini-Mental State Examination (MMSE), a common tool for screening cognitive impairment in different countries worldwide, including China (Li et al., 2016). The total score of 30 items is in the range of 0–30, with higher MMSE scores indicating better cognitive function (Katzman et al., 1988). Cognitive impairment was defined as a MMSE score less than education-adjusted normal value (17 points for illiterate, 20 points for primary school, and 24 points for junior high school and above) (Li et al., 2016).

### Assessment of mobility

TUGT results (time in seconds) indicate mobility at a fast self-paced walking speed (Podsiadlo and Richardson, 1991). This test was conducted to measure the amount of time of the participant’s back leaving the chair and the amounts of time standing up, walking 3 m as quickly and safely as possible, turning around, walking back to the chair, and sitting down again with the back against the chair. The task was demonstrated before participants were asked to complete it. The cut-off time of ≥20 s was indicative of impaired physical performance (Cruz-Jentoft et al., 2019).

### Assessment of muscle strength

In the present study, knee extension measurements were selected to characterize overall lower extremity muscle strength (Bohannon et al., 2012). The measurements were performed by the same principal investigator on the dominant side of each participant by using a dynamometer (S-03158B, SAKAImed Corp., Tokyo, Japan). Each participant sat on a chair whose legs were perpendicular to the ground and were secured to his or her calf with a seat belt. During isometric muscle force measurements, the dynamometer sensor was attached to the ankle with Velcro, the torso and thigh were stabilized, and the anchor strap was attached to a usable structure that directly opposes the knee extension movement. Before starting, the monitor was set in the OFFSET zeroing state to prevent the effect of gravity. The zeroing state was ensured by gently holding the monitor. The participants’ hands were adjusted on their chests, and they were asked to kick toward the floor until the maximum force was reached. This task was repeated, and the mean values of two tests of the right leg were used for subsequent analyses. The mean values of two tests of the right leg were used for analysis. Low muscle strength was defined as knee extension strength < 18 kg in men and < 16 kg in women (Assantachai et al., 2014).

### Covariates

Data including sociodemographic characteristics, behavioral performance, and medical conditions were obtained *via* face-to-face interviews. Sociodemographic variables included sex, age, education level, and occupation. Behavioral characteristics included drinking (daily, former, never), and smoking habits (daily, <7 days/week, former, never), and physical characteristics. Former drinkers were defined as those who used to drink frequently but did not drink for at least half a year. Former smokers were defined as those who were abstinent for over half a year. Body mass index (BMI) was calculated as the weight in kilograms divided by the height in meters squared and measured in accordance with the WHO BMI category (World Health Organization technical report series, 2000). Medical history, including stroke, diabetes, hypertension, and heart disease, was obtained from the responses of participants to questions about their history, past diagnoses made by physicians, and current or historical medication regimens. The Geriatric Depression Scale (GDS-30) was used to evaluate depression. Participants with scores ≥11 were considered to be mildly depressed, and those with scores ≥21 were considered to be moderately–severely depressed (Neal and Baldwin, 1994).

### Statistical analysis

All statistical analyses were performed using IBM SPSS Statistic v26.0 software (SPSS Inc., Chicago, IL, USA). Continuous variables with normal distribution are presented as the means and standard deviation (mean ± SD), and categorical variables are presented as percentages. The MMSE scores, TUGT time, and knee extension strength of the participants were compared using the independent sample t test for two categorical groups or analysis of variance for more than two categorical groups followed by Bonferroni post-hoc test. Bivariate correlations were determined by Pearson’s (for continuous variables) or Spearman’s (for categorical variables) coefficients. Independent effects of lower extremity muscle strength and mobility metrics on cognitive function were examined using linear regression analysis. Odds ratios and 95% confidence intervals (CIs) were computed.

Mediating analytic was statistically analyzed using PROCESS Model 4 developed by Hayes (Preacher and Hayes, 2004). The capital letters X, M, and Y were used to represent mobility, lower extremity muscle strength, and cognitive function, respectively. The mediating effect was tested in four pathways (regression coefficients): mobility on muscle strength (Path a), muscle strength on cognitive function (Path b), mobility on cognitive function without medication (Path c), mobility on cognitive function with medication (Path c’). The value of the mediating effect was calculated as a*b, and the ratio of the mediating effect to the total effect was a*b/c. The mediating effect value was examined using a bootstrap method to verify the mediating effect (a is the regression correlation coefficient of path a, b is the regression correlation coefficient of path b, c is the regression correlation coefficient of path c, and c’ is the regression correlation coefficient of path c’). Statistical significance of mediator variables was tested in over 5,000 bootstrap samples. The method generated an estimate of the indirect effect, including 95% CIs. As suggested by Hayes, we inferred whether the mediating effects were statistically significant based on 95% CI (excluding zero). Statistical significance was considered at *p* value <0.05.

## Results

### Effect of demographic variables on the MMSE scores, TUGT time, and knee extension strength of older adults

In total, 657 older adults aged over 65 years old were surveyed in this study, and 595 surveys were completed, yielding a response rate of 90.56%. The demographic and related characteristics and the MMSE, TUGT, and knee extension strength scores of the participants are presented in Tables 1–3. The average MMSE score, TUGT time, and knee extension strength were 24.75 ± 4.97, 8.63 ± 5.20, and 18.72 ± 8.36, respectively. Participants with different ages, education levels, jobs, drinking habits and GDS scores had significantly different MMSE scores, TUGT time, and knee extension strengths (*p* < 0.05). Different sex, job, and smoking habits can significantly affect MMSE score and knee extension strength (*p* < 0.05). The participants with varying BMIs had significantly different knee extension strengths (*p* < 0.05). Comparisons between groups were performed using post-hoc Bonferroni comparisons. Regarding age, *post hoc* analysis (see Supplementary Table S1) revealed that unsurprisingly, the performance of MMSE scores, TUGT time, and knee extension strength in older adults aged over 85 years old were significantly more likely to be worse than other age groups (details see in Supplementary Table S1). The all results of the Bonferroni *post hoc* test are in Supplementary Tables S1, S2.

**Table 1:** Differences of MMSE, TUGT and knee extension strength in demographic variables (*N* = 595).

Variables	Age (years)			*df*	*F*-value	*p*-value	Education			*df*	*F*-value	*p*-value	Job			*df*	*F*-value	*p*-value	BMI (kg/m^2^)				*df*	*F*- value	*P*- value
	65–74	75–84	≥85				Illiteracy	Primary school	Junior high school and above				Mental labor	Physical labor	Both				Underweight (<18·5)	Healthy weight (18·5–24·9)	Overweight (25·0–29·9)	Obese (≥30·0)			
N	413	162	20				69	299	227				83	457	55				13	280	256	46			
MMSE	25.55 ±4.41	23.62 ±4.85	17.30 ±8.31	592	35.75	0.000***	18.45 ±5.48	24.67 ±4.50	26.78 ±3.60	592	98.80	0.000***	26.61 ±4.31	24.27 ±5.16	25.93 ±3.23	592	9.80	0.000***	25.46 ±2.26	24.67 ±5.23	24.86 ±4.77	24.39 ± 5.08	591	0.24	0.872
TUGT (s)	7.69 ±2.48	9.83 ±7.40	18.41 ±10.79	592	54.70	0.000***	11.67 ±11.14	8.32 ±3.08	8.12 ±4.25	592	14.00	0.000***	9.78 ±1.03	8.42 ±4.03	8.64 ±5.20	592	2.38	0.09	9.29 ±4.77	8.94 ±6.38	8.04 ±2.61	9.98 ± 7.47	591	2.41	0.066
Knee extension strength (kg)	19.79 ±8.29	16.84 ±8.20	11.82 ±4.80	592	14.94	0.000***	15.00 ±6.01	18.38 ±8.07	20.29 ±8.96	592	11.50	0.000***	18.28 ± 8.16	20.88 ± 9.74	19.68 ± 8.31	592	3.04	0.05	15.00 ± 9.58	17.54 ± 7.49	20.11 ± 8.91	19.20 ± 8.84	591	5.24	0.001

Values were expressed as mean ± SD; analysis of variance was performed; P value < 0.05 was considered as statistical significance, ***p < 0.001; BMI, Body Mass Index; df, degree of freedom; MMSE, Mini-Mental State Examination; TUGT, Timed Up-and-Go Test.

**Table 2:** Differences of MMSE, TUGT and knee extension strength in demographic variables (*N* = 595).

Variables	Drinking				*df*	*F*-value	*p*-value	Smoking			*df*	*F*-value	*p*-value	GDS			*df*	*F*-value	*p*-value
	Daily	<7 Days/week	Former	Never				Daily	Former	Never				Normal	Mild	Moder-sever			
N	97	76	55	367				77	92	426				500	88	7			
MMSE	26.01 ± 3.50	25.05 ± 4.82	24.51 ± 4.63	24.39 ± 5.32	591	2.88	0.039	25.53 ± 4.26	25.96 ± 3.30	24.35 ± 5.33	592	5.14	0.006	24.88 ± 4.96	24.41 ± 4.57	19.43 ± 7.81	592	4.45	0.012
TUGT (s)	7.55 ± 1.77	8.06 ± 2.20	10.95 ± 10.88	8.69 ± 4.84	591	5.61	0.001	7.86 ± 2.39	9.36 ± 8.10	8.61 ± 4.74	592	1.78	0.169	8.38 ± 4.78	9.29 ± 4.24	18.28 ± 19.93	592	13.91	0.000***
Knee extension strength (kg)	22.26 ± 8.58	20.08 ± 8.78	20.42 ± 8.57	17.24 ± 7.83	591	10.96	0.000***	22.36 ± 9.02	20.91 ± 9.46	17.59 ± 7.70	592	15.02	0.000***	19.02 ± 8.49	17.69 ± 7.36	10.01 ± 5.57	592	4.84	0.008

Values were expressed as mean ± SD; analysis of variance was performed; P value < 0.05 was considered as statistical significance, ***P < 0.001; df, degree of freedom; GDS, Geriatric Depression Scale; MMSE, Mini-Mental State Examination; TUGT, Timed Up-and-Go Test.

**Table 3:** Differences of MMSE, TUGT, and knee extension strength in demographic variables (*N* = 595).

Variables	Sex		*df*	*T*-value	*p*-value	Live alone		*df*	*T*-value	*p*-value	Stroke		*df*	*T*-value	*p*-value	Diabetes		*df*	*T*-value	*p*-value	Hypertension		*df*	*T*-value	*p*-value	Heart Disease		*df*	*T*-value	*p*-value
	Male	Female				Yes	No				Yes	No				Yes	No				Yes	No				Yes	No			
N	243	352				76	519				152	443				93	502				382	213				171	424			
MMSE	25.89 ± 3.37	23.86 ± 5.70	593	4.73	0.000***	23.53 ± 4.46	24.93 ± 5.02	593	−2.31	0.021	24.32 ± 5.03	24.90 ± 4.95	593	−1.23	0.22	24.17 ± 5.43	24.86 ± 4.88	593	−1.22	0.223	24.64 ± 4.83	24.95 ± 5.22	593	−0.73	0.467	23.87 ± 5.59	25.10 ± 4.66	593	−2.75	0.006
TUGT (s)	8.56 ± 5.56	8.69 ± 4.95	593	−0.32	0.767	9.80 ± 8.02	8.47 ± 4.63	593	2.11	0.036	9.69 ± 6.72	8.27 ± 4.52	593	2.89	0.004	9.26 ± 5.62	8.52 ± 5.12	593	1.27	0.208	8.76 ± 4.96	8.40 ± 5.61	593	0.80	0.416	9.31 ± 6.56	8.36 ± 4.52	593	2.02	0.045
Knee extension strength (kg)	22.44 ± 9.66	16.15 ± 6.15	593	9.69	0.000***	17.61 ± 7.59	18.88 ± 8.47	593	−1.24	0.216	16.94 ± 7.21	19.33 ± 8.65	593	−3.06	0.002	18.77 ± 7.97	18.71 ± 8.44	593	0.07	0.946	18.98 ± 8.26	18.30 ± 8.57	593	0.92	0.359	17.83 ± 8.16	19.07 ± 8.43	593	0.54	0.100

Values were expressed as mean ± SD; *t* test for two categorical groups was performed; *p* value < 0.05 was considered as statistical significance; GDS, Geriatric Depression Scale; MMSE, Mini-Mental State Examination; TUGT, Timed Up-and-Go Test.

### Correlations between MMSE scores, TUGT time, knee extension strength, and other covariates

The MMSE scores were moderately correlated with the TUGT time (r = −0.315, *p* < 0.01; see in Table 4), but weakly correlated with the knee extension strength (r = 0.286, *p* < 0.01; see in Table 4). In addition, the TUGT time was weakly correlated with knee extension strength (r = −0.272, *p* < 0.01; see in Table 4). Moreover, the MMSE scores were moderately correlated with the education (r = 0.465, *p* < 0.01), while weakly correlated with age, sex, GDS, job, drinking habit and living status (detail see in Tables 4, 5). The TUGT time was weakly correlated with age, GDS, the education and living status (detail see in Tables 4, 5), and knee extension strength was weakly correlated with age, sex, BMI, GDS, the education and drinking habits (details see in Tables 4, 5).

**Table 4:** Correlation between MMSE, TUGT, knee extension strength, and other covariates.

Variables	MMSE	TUGT	knee extension strength	Age	BMI	GDS
MMSE	1	−0.315***	0.286***	−0.217***	−0.033	−0.103*
TUGT	–	1	−0.272***-	0.264***	0.017	0.152***
knee extension strength	–	–	1	−0.178***	0.186***	−0.115**

Pearson’s correlation analysis; *p < 0.05, **p < 0.01, ***p < 0.001; BMI, Body Mass Index; GDS, Geriatric Depression Scale; MMSE, Mini-Mental State Examination; TUGT, Timed Up-and-Go Test.

**Table 5:** Correlation between MMSE, TUGT, knee extension strength, and other covariates.

Variables	Sex (male/female)	Education (illiteracy/primary school/junior high school and above)	Job (mental labor/Physical labor/Both)	Drinking (daily/<7 Days/week/former/never)	Smoking (daily/former/never)	Live alone (Yes/No)
MMSE	−0.143***	0.465***	−0.118**	−0.097*	0.030	0.146***
TUGT	0.047	−0.222***	−0.024	0.075	0.027	−0.138**
knee extension strength	−0.346***	0.176***	0.001	−0.231***	−0.037	0.054

Spearman’s correlation analysis; *p < 0.05, **p < 0.01, ***p < 0.001; MMSE, Mini-Mental State Examination; TUGT, Timed Up-and-Go Test.

### Risk factors for cognitive function

After adjusting for potential confounders in model 3 (i.e., sex, age, education, job, BMI, GDS, living status, drinking habits, smoking habits, and comorbidity status), the two factors for cognitive function were summarized below: TUGT (adjusted hazard ratio, [HR] = −0.24; 95% CI = -0.33--0.14, *p* < 0.001; see in Table 6), knee extension strength (adjusted HR = 0.05; 95% CI =0.00–0.10, *p* = 0.03; see in Table 6). The linear regression results for all the variables were shown in Supplementary Tables S1–S3.

**Table 6:** Linear regression analysis of TUGT and knee extension strength on cognition function.

Variables	Unadjusted		Model 1		Model 2		Model 3	
	Hazard ratio (95% CI)	*P*-value	Hazard ratio (95% CI)	*P*-value	Hazard ratio (95% CI)	*P*-value	Hazard ratio (95% CI)	*P*-value
TUGT	−0.25 (−0.32, −0.17)	0.000***	−0.16 (−0.23, −0.08)	0.000***	−0.23 (−0.32, −0.14)	0.000***	−0.24 (−0.33, −0.14)	0.000***
knee extension strength	0.13 (0.08, 0.18)	0.000***	0.06 (0.01, 0.11)	0.01	0.06 (0.01, 0.11)	0.03	0.05 (0.00, 0.10)	0.03

Linear regression analysis was performed; *p* value < 0.05 was considered as statistical significance, *** *p* < 0.001; Model 1: sex, age, education and job; Model 2: Model 1 + BMI, GDS, living status, drinking and smoking habits; Model 3: Model 2+ comorbidity status (stroke, diabetes, hypertension, and heart diseases); TUGT, Timed Up-and-Go Test.

### Analysis of the mediating role of lower extremity muscle strength between mobility and cognition function

**Figure 1:**
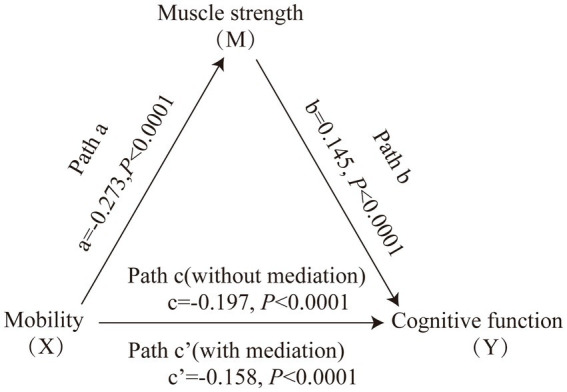
Mediating role of muscle strength on the relationships between mobility and cognitive function. The capital letters X, M, and Y were used to represent mobility, lower extremity muscle strength and cognitive function, respectively. The mediating effect tested the four following pathways (regression coefficients): mobility on muscle strength (Path a), muscle strength on cognitive function (Path b), mobility on cognitive function without medication (Path c), mobility on cognitive function with medication (Path c’).

**Figure 2:**
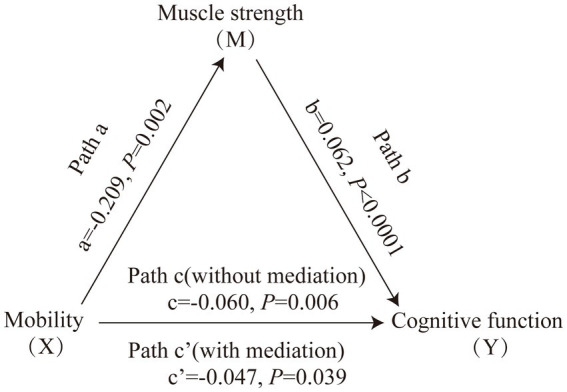
Mediating role of muscle strength on the relationships between mobility and cognitive function in community-dwelling older adults with normal cognition. The capital letters X, M, and Y were used to represent mobility, lower extremity muscle strength and cognitive function, respectively. The mediating effect tested the four following pathways (regression coefficients): mobility on muscle strength (Path a), muscle strength on cognitive function (Path b), mobility on cognitive function without medication (Path c), mobility on cognitive function with medication (Path c’).

**Figure 3:**
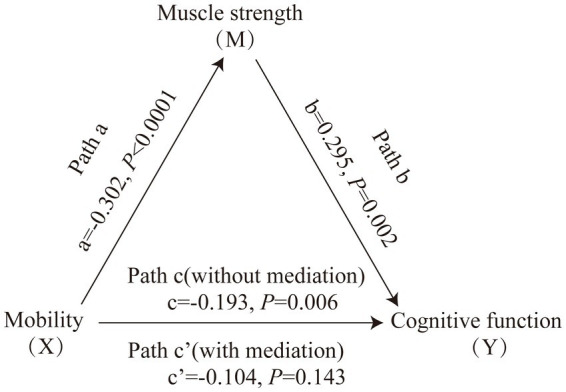
Mediating role of muscle strength on the relationships between mobility and cognitive function in community-dwelling older adults with cognitive impairment. The capital letters X, M, and Y were used to represent mobility, lower extremity muscle strength and cognitive function, respectively. The mediating effect tested the four following pathways (regression coefficients): mobility on muscle strength (Path a), muscle strength on cognitive function (Path b), mobility on cognitive function without medication (Path c), mobility on cognitive function with medication (Path c’).

Figure 1 displays the mediating role of lower extremity muscle strength on the relationship between mobility and cognition function (Table 7). After controlling for sociodemographic and related variables, a significant total effect of mobility on cognitive function was identified (path c: c = −0.197, *p* < 0.001). In path a, mobility had a negative impact on muscle strength (path a: a = −0.273, *p* < 0.001). In path b, muscle strength had a positive impact on cognitive function (path b: b = 0.145, *p* < 0.001). In addition, mobility had a negative impact on cognitive function (path c’: c’ = −0.158, *p* < 0.001). The mediating effect value was calculated as 0.273*0.145, that is, 0.039, and the ratio of the mediating effect over the total effect was 20.1% (0.039/0.197 = 0.201). A summary of the mediating role of lower extremity muscle strength in the relationship between mobility and cognition function is shown in Table 7. In addition, the mediating effect test was conducted using the bootstrap method with 5,000 samples. Results showed that the 95% CI of the mediating effect value of lower extremity muscle strength did not include zero (95% CI: −0.0749, −0.0293, *p* < 0.001), indicating that lower extremity muscle strength exerted a significant mediating effect on the relationship between mobility and cognition function. The regression correlation coefficients of paths a, b, c, and c’ were all significant. Considering that the different trends of declining in muscle strength at various cognitive stages, the population was categorized into normal cognitive (n = 503) and cognitive impaired (*n* = 92) groups by education-adjusted MMSE cut-off score (16/17 for illiterate, 19/20 for primary school, and 23/24 for junior high school and above) (Li et al., 2016) for separately mediating effects analyses. The similar results were found in community-dwelling older adults with normal cognition (see Supplementary Table S4 and Figure 2), but not in ones with cognitive impairment (see Supplementary Table S5 and Figure 3), whose regression correlation coefficient of paths c’ was not significant (*p* = 0.143).

**Table 7:** Summary of the mediating effects of lower extremity muscle strength between mobility and cognitive function.

Effect	Independent variables	Dependent variables	*B*	*SE*	*t*	*p* value	95%CI
Total effect (c)	X	Y	−0.197	0.031	−6.406	0.000***	(−0.258, −0.137)
Indirect effect (a)	X	M	−0.273	0.052	−5.206	0.000***	(−0.376, −0.170)
Indirect effect (b)	M	Y	0.145	0.023	6.176	0.000***	(0.099, 0.191)
Direct effect (c’)	X	Y	−0.197	0.031	−6.406	0.000***	(−0.258, −0.137)

Mediating analytic was statistically analyzed; ***p < 0.001; B, unstandardized coefficient; SE, standard error; X, mobility; M, lower limb muscle strength; Y, cognitive function.

## Discussion

In this study, poor cognitive function was moderately associated with poor mobility, and muscle strength was weakly correlated with cognitive function and mobility, respectively. Sex, age, education, job, GDS, drinking and smoking habits, living status and heart disease were identified as the potential influencing factors of cognitive function in this study. And age, education, job, GDS, drinking habit, living status, stroke and heart disease were identified as the potential influencing factors of mobility, while sex, age, education, job, BMI, GDS, drinking and smoking habits, and stroke were identified as the potential influencing factors of muscle strength in this study. Some previous studies identified similar influencing factors in older adults (Steffen et al., 2002; Germain et al., 2016; Jia et al., 2020). After adjusting above potential confounding factors, the results of linear regression analysis showed that mobility and muscle strength were the independent factors of cognitive function. Consequently, muscle strength significantly affected the partially mediation of the association between mobility and cognitive function, and the mediating effect value was 20.1%.

To the best of our knowledge, this is the first cross-sectional study investigating the influence of lower extremity muscle strength on the relationship among mobility and cognitive function in Chinese older adults. In previous studies considering the association of physical performance and cognitive function, both grip strength (McGrath et al., 2020; Filardi et al., 2022) and chair stand test (Dodds et al., 2021) have been extensively used as measures of muscle strength. However, compared to grip strength (*r* = −0.35), lower-extremity muscle strength correlations with gait speed were higher (*r* = −0.49) indicating that lower-extremity muscle strength may be more direct measures of the muscle groups needed to complete the gait test, whereas grip strength may be a proxy measure of lower-extremity muscle strength (Winger et al., 2021). Additionally, chair stand test measures the time needed to rise from a chair and sit down again five times without using the arms, which represents dynamic weight-bearing lower extremity muscle function (not just muscle strength) (Guralnik et al., 1994). It requires more complex muscle movement coordination (Winger et al., 2021) and overlaps with the starting movement of the TUGT test (Podsiadlo and Richardson, 1991), which could be potential confounding factor for the association of physical performance and cognitive function. This suggests that knee extension strength may be more appropriate than chair stand test, reflects immediate muscle strength in the absence of weight bearing, and more simply and directly reflects the potential correlation between muscle strength and TUGT (Benavent-Caballer et al., 2016).

Previous studies (Liu et al., 2021; Wu et al., 2021) reported that mobility and muscle strength are correlated with cognitive function, which is consistent with the findings of the present study. The potential biological pathways for this relationship are being investigated, and several potential mechanisms have been proposed. Growing studies of age-related skeletal muscle dysfunction concerns the combination of neural and muscular factors (Aubertin-Leheudre et al., 2020; Coletti et al., 2022). The neuromuscular junction (NMJ), as the intersection between the nervous and muscular systems, couples these systems for motor output and control and forms a link for bidirectional communication (Castets et al., 2020; Padilla et al., 2021). Muscle strength primarily decreases with aging because of alpha motoneuron loss, NMJ dysfunction, and the subsequent muscle fiber denervation and motor unit loss (Doherty et al., 1993; Padilla et al., 2021). Moreover, lower muscle strength has been associated with increased reaction time (Jiménez-García et al., 2021), hence directly adding the scored time of mobility during TUGT. Age-related decline in muscle strength precedes the decline of mobility in older adults and then impairs performance in activities of daily living (Clark et al., 2013), whereas decreased physical activity aggravates NMJ degeneration and results in nerve terminal sprouting (Coletti et al., 2022). Given that peripheral nerves interact with skeletal muscles at the NMJ, modifications of this bidirectional communication by mobility are related to brain glucose metabolism in specific regions, such as the entorhinal cortex (Shimada et al., 2017). The entorhinal cortex is part of a key pathway for memory formation and is responsible for receiving afferents from broadly associative and limbic regions and sending afferents back to the association neocortex and the dentate gyrus of the hippocampus (Zola-Morgan et al., 1994). Connections between the cerebellum and hippocampus are involved as another potential mechanism in early concomitant age-related physical and cognitive decline (Liu et al., 2020), and they contribute to understanding the ageing basis of the cerebellum in physical performance and cognitive function (Bernard and Seidler, 2014). Understanding the role of muscle strength in the pathway to physio-cognitive decline is important for identifying effective strategies to delay the onset of age-related physio-cognitive dysfunction.

Furthermore, the results of the mediation effect analysis of cognitive impaired group in our study showed a non-significant direct effect, indicating muscle strength was the key mediator of cognitive function and mobility not just partially. It may be due to a bias caused by the small sample size (n = 92), or the fact that patients with cognitive impairment show a significant decrease in muscle mass during the progression of cognitive decline (Ogawa et al., 2018), further reinforcing the correlation with muscle strength in cognitive impairment patients (Filardi et al., 2022). Future studies with larger sample size are needed to validate the potential mechanism in the cognitive impairment population.

This study has some limitations. To begin with, it is a cross-sectional study that was unable to determine causality, and further studies with longitudinal follow-up and larger sample sizes are needed to determine the reliability and validity of this model. In addition, participants in this study were relatively less restricted in their mobility. In the overall ageing population, the true decline trend may be in a greater degree. The power of the model is possibly underestimated. Since this study did not observe the older adults with dementia, the conclusions cannot be applied to these individuals. Previous studies have found that impairments in non-memory cognitive domains, such as executive and language function, begin much earlier than memory deficits (van der Leeuw et al., 2016). Therefore, different cognitive fields must be considered in future research.

## Conclusion

Lower extremity muscle strength exerts a partially significant mediating effect on the relationship between mobility and cognitive function.

## Data availability statement

The original contributions presented in the study are included in the article/Supplementary material, further inquiries can be directed to the corresponding author.

## Ethics statement

The studies involving human participants were reviewed and approved by the Ethics Committee of Shanghai University of Medicine and Health Sciences (No. 2018-E4-6100-18-201067-03-210302197009090947). The patients/participants provided their written informed consent to participate in this study.

## Author contributions

JP and HW: study concept and design. YCh, YZ, HZ, YCa, LW, WZ, and HS: investigation and acquisition and evaluation of data. YCh: drafting of the manuscript. YCh and YZ: analysis of data. YZ: writing-reviewing and editing. JP: funding acquisition and project administration. All authors contributed to the article and approved the submitted version.

## Funding

This project was supported by the grant of the Key Scientific Research Program of Shanghai Municipal Science and Technology Committee in China (18401970500 and 22Y11922900), the TCM genre program of Shanghai Health Bureau [ZY (2018-2020)-CCCX-1006 and ZY (2021-2023)-0209-10], and Clinical Research Plan of SHDC (SHDC2020CR3091B).

## Conflict of interest

The authors declare that the research was conducted in the absence of any commercial or financial relationships that could be construed as a potential conflict of interest.

## Publisher’s note

All claims expressed in this article are solely those of the authors and do not necessarily represent those of their affiliated organizations, or those of the publisher, the editors and the reviewers. Any product that may be evaluated in this article, or claim that may be made by its manufacturer, is not guaranteed or endorsed by the publisher.
