# Supplementary material for: Mediating effect of lower extremity muscle strength on the relationship between mobility and cognitive function in Chinese older adults: A cross-sectional study
Source: Front Aging Neurosci. 2022 Nov 3;14:984075. doi: 10.3389/fnagi.2022.984075 (PMC9669366; doi:10.3389/fnagi.2022.984075)
Supplement: Supplementary file 1 [file Data_Sheet_1.docx]

Supplementary Table S1: One-way ANOVA with Bonferroni Post-Hoc Test for Table 1

| Variables | **df** | | F  value | **Age (years)** | | |  | **df** | | F  value | **Education** | | |  | **df** | | F  value | **JOB** | | |  | **df** | | F  value | **BMI (**kg/m^2^**)** | | | | | |
| --- | --- | --- | --- | --- | --- | --- | --- | --- | --- | --- | --- | --- | --- | --- | --- | --- | --- | --- | --- | --- | --- | --- | --- | --- | --- | --- | --- | --- | --- | --- |
|  |  |  |  | 65-74  vs  75-84 | 75-84  vs  ≥85 | 65-74  vs  ≥85 |  |  |  |  | Illiteracy  vs  Primary school | Illiteracy  vs  Junior high school and above | Primary school  vs  Junior high school and above |  |  |  |  | Mental labor  vs  Physical labor | Physical labor  vs  Both | Mental labor  vs  Both |  |  |  |  | Underweight  vs  Healthy weight | Healthy weight  vs  Overweight | Underweight  vs  Overweight | Underweight  vs  Obese | Healthy weight  vs  Obese | Overweight  vs  Obese |
|  | BG | WG |  | *P* | *P* | *P* |  | BG | WG |  | *P* | *P* | *P* |  | BG | WG |  | *P* | *P* | *P* |  | BG | WG |  | *P* | *P* | *P* | *P* | *P* | *P* |
| MMSE | 2 | 592 | 35.75 | 0.000*** | 0.000*** | 0.000*** |  | 2 | 592 | 98.80 | 0.000*** | 0.000*** | 0.000*** |  | 2 | 592 | 9.80 | 0.000*** | 0.054 | 1.000 |  | 3 | 591 | 0.24 | 1.000 | 1.000 | 1.000 | 1.000 | 1.000 | 1.000 |
| TUGT (s) | 2 | 592 | 54.70 | 0.000*** | 0.000*** | 0.000*** |  | 2 | 592 | 14.00 | 0.000*** | 0.000*** | 1.000 |  | 2 | 592 | 2.38 | 0.086 | 1.000 | 0.612 |  | 3 | 591 | 2.41 | 1.000 | 0.275 | 1.000 | 1.000 | 1.000 | 0.156 |
| Knee extension strength (kg) | 2 | 592 | 14.94 | 0.000*** | 0.029 | 0.000*** |  | 2 | 592 | 11.50 | 0.007 | 0.000*** | 0.025 |  | 2 | 592 | 3.04 | 0.476 | 0.088 | 1.000 |  | 3 | 591 | 5.24 | 1.000 | 0.002 | 0.182 | 0.638 | 1.000 | 1.000 |

Note: analysis of variance for more than two categorical groups followed by Bonferroni post-hoc test; Abbreviation: BG, between group; df, degree of freedom; WG, within group; *P*, p-value between groups; *P* value < 0.05 was considered as statistical significance, ****P* < 0.001;

Supplementary Table S2: One-way ANOVA with Bonferroni Post-Hoc Test for Table 2

| Variables | **df** | | F  value | **Drinking** | | | | | |  | **df** | | F  value | **Smoking** | | |  | **df** | | F  value | **GDS** | | |  |
| --- | --- | --- | --- | --- | --- | --- | --- | --- | --- | --- | --- | --- | --- | --- | --- | --- | --- | --- | --- | --- | --- | --- | --- | --- |
|  |  |  |  | Daily  vs  <7 Days/week | <7 Days/week  vs  Former | Daily  vs  Former | Daily  vs  Never | <7 Days/week  vs  Never | Former  vs  Never |  |  |  |  | Daily  vs  Former | Former  vs  Never | Daily  vs  Never |  |  |  |  | Normal  vs  Mild | Mild  vs  Moder-sever | Normal  vs  Moder-sever |  |
|  | BG | WG |  | *P* | *P* | *P* | *P* | *P* | *P* |  | BG | WG |  | *P* | *P* | *P* |  | BG | WG |  | *P* | *P* | *P* |  |
| MMSE | 3 | 591 | 2.88 | 1.000 | 1.000 | 0.373 | 0.029 | 1.000 | 1.000 |  | 2 | 592 | 5.14 | 1.000 | 0.015 | 0.177 |  | 2 | 592 | 4.45 | 1.000 | 0.032 | 0.012 |  |
| TUGT (s) | 3 | 591 | 5.61 | 1.000 | 0.008 | 0.000*** | 0.000*** | 1.000 | 0.010 |  | 2 | 592 | 1.78 | 0.168 | 0.512 | 0.801 |  | 2 | 592 | 13.91 | 0.374 | 0.000*** | 0.000*** |  |
| Knee extension strength (kg) | 3 | 591 | 10.96 | 0.487 | 1.000 | 1.000 | 0.000*** | 0.049 | 0.057 |  | 2 | 592 | 15.02 | 0.757 | 0.002 | 0.000*** |  | 2 | 592 | 4.84 | 0.497 | 0.057 | 0.014 |  |

Note: analysis of variance for more than two categorical groups followed by Bonferroni post-hoc test; Abbreviation: BG, between group; df, degree of freedom; WG, within group; *P*, p-value between groups;

*P* value < 0.05 was considered as statistical significance, ***P < 0.001;
