# Supplementary material for: Mediating effect of lower extremity muscle strength on the relationship between mobility and cognitive function in Chinese older adults: A cross-sectional study
Source: Front Aging Neurosci. 2022 Nov 3;14:984075. doi: 10.3389/fnagi.2022.984075 (PMC9669366; doi:10.3389/fnagi.2022.984075)
Supplement: Supplementary file 2 [file Data_Sheet_2.docx]

Supplementary Table S3: Linear regression analysis of covariates on cognition function in Model 1

| Variables | | | |  | Model 1 | |  | Model 2 | |  | Model 3 | |
| --- | --- | --- | --- | --- | --- | --- | --- | --- | --- | --- | --- | --- |
|  |  |  |  |  | Hazard ratio (95% CI) | *P* value |  | Hazard ratio (95% CI) | *P* value |  | Hazard ratio  (95% CI) | *P* value |
| Sex | Female | | |  | 1 |  |  | 1 |  |  | 1 |  |
|  | Male | | |  | 0.71  (-0.06, 1.48) | 0.071 |  | 0.69  (-0.42, 1.80) | 0.221 |  | 0.75  (-0.09, 2.20) | 0.071 |
| Age |  | | |  | -0.15  (-0.22, -0.09) | 0.000*** |  | -0.10  (-0.17, -0.04) | 0.002 |  | -0.10  (-0.17, -0.04) | 0.003 |
| Education | | Illiteracy | |  | 1 |  |  | 1 |  |  | 1 |  |
|  |  | Primary school | |  | 4.18  (3.13, 5.23) | 0.000*** |  | 4.77  (3.74, 5.80) | 0.000*** |  | 4.79  (3.75, 5.82) | 0.000*** |
|  |  | Junior high school and above | |  | 5.51  (4.35, 6.68) | 0.000*** |  | 6.27  (-0.32, -0.14) | 0.000*** |  | 6.23  (5.09, 7.38) | 0.000*** |
| Job | | | Mental labor |  | 1 |  |  | 1 |  |  | 1 |  |
|  |  |  | Physical labor |  | -1.25  (-2.31, -0.18) | 0.021 |  | -1.16  (-2.24, -0.08) | 0.000*** |  | -1.18  (-2.26, -0.09) | 0.034 |
|  |  |  | Both |  | -0.66  (-2.09, 0.77) | 0.37 |  | -0.47  (-1.89,0.96) | 0.523 |  | -0.46  (-1.90, 0.61) | 0.311 |

Notes: Linear regression analysis was performed; *P* value < 0.05 was considered as statistical significance, *** *P* < 0.001; Model 1: sex, age, education and job; Model 2: Model 1+BMI, GDS, living status, drinking and smoking habits; Model 3: Model 2+ comorbidity status (stroke, diabetes, hypertension and heart diseases); BMI, Body mass index; GDS, The Geriatric Depression Scale

Supplementary Table S4: Linear regression analysis of covariates on cognition function in Model 2

| Variables | | | |  | Model 1 | |  | Model 2 | |  | Model 3 | |
| --- | --- | --- | --- | --- | --- | --- | --- | --- | --- | --- | --- | --- |
|  |  |  |  |  | Hazard ratio (95% CI) | *P* value |  | Hazard ratio (95% CI) | *P* value |  | Hazard ratio  (95% CI) | *P* value |
| BMI |  | | |  | - | - |  | 0.00  (-0.08, 0.09) | 0.970 |  | 0.02  (-0.07, 0.10) | 0.686 |
| GDS |  | | |  | - | - |  | -0.03  (-0.11, 0.05) | 0.486 |  | -0.02  (-0.10, 0.06) | 0.620 |
| Live alone | | Yes | |  | - | - |  | 1 |  |  | 1 |  |
|  |  | No | |  | - | - |  | 0.04  (-0.96, 1.03) | 0.944 |  | 0.01  (-0.99, 1.01) | 0.982 |
| Drinking | | | Never |  | - | - |  | 1 |  |  | 1 |  |
|  |  |  | <7 Days/week |  | - | - |  | -0.88  (-1.91, 0.95) | 0.098 |  | -0.88  (-1.92, -0.16) | 0.098 |
|  |  |  | Former |  | - | - |  | -0.77  (-2.02, 0.48) | 0.227 |  | -0.74  (-2.00, 0.52) | 0.249 |
|  | | | Daily |  | - | - |  | -0.07  (-1.08, 0.95) | 0.889 |  | -0.06  (-1.08, 0.96) | 0.903 |
| Smoking | | | Never |  | - | - |  | 1 |  |  | 1 |  |
|  |  |  | Former |  | - | - |  | 0.10  (-1.11, 1.32) | 0.870 |  | 0.15  (-1.07, 1.37) | 0.812 |
|  |  |  | Daily |  | - | - |  | -0.59  (-1.88, 0.66) | 0.355 |  | -0.65  (-1.90, 0.97) | 0.527 |

Notes: Linear regression analysis was performed; *P* value < 0.05 was considered as statistical significance; Model 1: sex, age, education and job; Model 2: Model 1+BMI, GDS, living status, drinking and smoking habits; Model 3: Model 2+ comorbidity status (stroke, diabetes, hypertension and heart diseases); BMI, Body mass index; GDS, The Geriatric Depression Scale

Supplementary Table S5: Linear regression analysis of covariates on cognition function in Model 3

| Variables | |  | Model 1 | |  | Model 2 | |  | Model 3 | |
| --- | --- | --- | --- | --- | --- | --- | --- | --- | --- | --- |
|  |  |  | Hazard ratio (95% CI) | *P* value |  | Hazard ratio (95% CI) | *P* value |  | Hazard ratio  (95% CI) | *P* value |
| Stroke | Yes |  | - | - |  | - | - |  | 1 |  |
|  | No |  | - | - |  | - | - |  | -0.18  (-0.97, 0.61) | 0.660 |
| Diabetes | Yes |  | - | - |  | - | - |  | 1 |  |
|  | No |  | - | - |  | - | - |  | 0.32  (-0.59, 1.23) | 0.488 |
| Hypertension | Yes |  | - | - |  | - | - |  | 1 |  |
|  | No |  | - | - |  | - | - |  | 0.46  (-0.26, 1.18) | 0.208 |
| Heart Diseases | Yes |  | - | - |  | - | - |  | 1 |  |
|  | No |  | - | - |  | - | - |  | 0.35  (-0.40, 1.11) | 0.358 |

Notes: Linear regression analysis was performed; *P* value < 0.05 was considered as statistical significance, *** *P* < 0.001; Model 1: sex, age, education and job; Model 2: Model 1+BMI, GDS, living status, drinking and smoking habits; Model 3: Model 2+ comorbidity status (stroke, diabetes, hypertension and heart diseases); BMI, Body mass index; GDS, The Geriatric Depression Scale

Supplementary Table S6: Summary of the mediating effects of lower extremity muscle strength between mobility and cognitive function in community-dwelling older adults with normal cognition (N=503).

| Effect | Independent variables | Dependent variables | B | SE | t | *P* value | 95%CI |
| --- | --- | --- | --- | --- | --- | --- | --- |
| Total effect(c) | X | Y | -0.060 | 0.022 | -2.737 | 0.006 | (-0.104, -0.017) |
| Indirect effect(a) | X | M | -0.209 | 0.066 | -3.175 | 0.002 | (-0.376, -0.170) |
| Indirect effect(b) | M | Y | 0.062 | 0.015 | 4.206 | 0.000*** | (0.099, 0.191) |
| Direct effect (c') | X | Y | -0.047 | 0.022 | -2.164 | 0.039 | (-0.090, -0.004) |

Note: Mediating analytic was statistically analyzed; *** *P* < 0.001; B, unstandardized coefficient; SE, standard error; X, mobility; M, lower limb muscle strength; Y, cognitive function

Supplementary Table S7: Summary of the mediating effects of lower extremity muscle strength between mobility and cognitive function in community-dwelling older adults with cognitive impairment (N=92).

| Effect | Independent variables | Dependent variables | B | SE | t | *P* value | 95%CI |
| --- | --- | --- | --- | --- | --- | --- | --- |
| Total effect(c) | X | Y | -0.193 | 0.068 | -2.825 | 0.006 | (-0.328, -0.057) |
| Indirect effect(a) | X | M | -0.302 | 0.076 | -3.968 | 0.000*** | (-0.453, -0.151) |
| Indirect effect(b) | M | Y | 0.295 | 0.090 | 3.285 | 0.002 | (0.116, 0.473) |
| Direct effect (c') | X | Y | -0.104 | 0.070 | -1.477 | 0.143 | (-0.243, 0.036) |

Note: Mediating analytic was statistically analyzed; ****P* < 0.001; B, unstandardized coefficient; SE, standard error; X, mobility; M, lower limb muscle strength; Y, cognitive function
